# TYK2 Deficiency Presenting as Refractory Disseminated BCG/Tuberculosis Infection in a Kazakh Child: A Case Report with Genetic Confirmation

**DOI:** 10.3390/genes16121445

**Published:** 2025-12-02

**Authors:** Nurgul Sikhayeva, Svetlana Volodchenko, Elena Kovzel, Aiganym Toleuzhanova, Aliya Romanova, Gulnar Tortayeva, Yelena Sagandykova, Marina Morenko, Aidos Bolatov, Ilyas Akhmetollayev

**Affiliations:** 1 National Center for Biotechnology, Korgalzhyn Highway 13/5, Astana 010000, Kazakhstan; aiga1999nym@gmail.com (A.T.); romanovaaliya@gmail.com (A.R.); iliyas@mail.ru (I.A.); 2 National Holding «QazBioPharm», Korgalzhyn Highway 13/5, Astana 010000, Kazakhstan; 3 “University Medical Center” Corporate Fund, St. Kerey, Zhanibek Khandar Khanov 5/1, Astana 010000, Kazakhstan; svetlanasv888@mail.ru (S.V.); elena.kovzel@umc.org.kz (E.K.); gulnart@bk.ru (G.T.); l.e.n.a.78@mail.ru (Y.S.); 4 Department of Pediatrics with Courses in Allergology, Immunology, Hematology, and Endocrinology, School of Medicine, “Medical University of Astana” NAO, Beibitshilik Street 49/A, Astana 010000, Kazakhstan; morenko_m.a@mail.ru; 5 School of Medicine, Shenzhen University, 3688 Nanhai Road, Shenzhen 518060, China; bolatovaidos@gmail.com; 6 Division of Strategic Development and Science, “Human Research & Development” LLP, Qabanbay Batyr Ave. 11/2, Astana 010000, Kazakhstan

**Keywords:** *TYK2*, primary immunodeficiency, BCG vaccine, tuberculosis, chronic granulomatous disease, whole-exome sequencing, Kazakhstan

## Abstract

**Background/Objectives:** Hereditary anomalies in the *TYK2* gene are the basis of a rare primary immunodeficiency, immunodeficiency-35, typified by an augmented vulnerability to mycobacterial and viral infections. Clinical overlap with chronic granulomatous disease (CGD) and other granulomatous disorders complicates diagnosis, particularly in nations where universal BCG vaccination is instituted. We present a pediatric case from Kazakhstan to broaden the clinical and molecular spectrum of *TYK2*-related immunodeficiency and accentuate diagnostic challenges. **Methods:** The proband underwent clinical assessment, immunophenotyping, and biochemical analysis during episodes of active pathology and subsequent follow-up. Whole-exome sequencing (WES) was executed, followed by confirmatory Sanger sequencing and segregation analysis in first-degree kin. Functional assays for phagocyte oxidative burst and phagocytosis were conducted to exclude CGD. **Results:** WES identified two rare *TYK2* variants (*c.209_212del*, pathogenic; *c.2395G>A*, previously reported as pathogenic in a Chinese patient with *TYK2* deficiency) and a heterozygous *MEFV* duplication (*c.761_764dup*). Paternal DNA was unavailable; therefore, allelic phase could not be formally established, but the combined genotype and phenotype are consistent with autosomal recessive *TYK2* deficiency. Sanger sequencing confirmed segregation of the frameshift *TYK2* variant in the mother, while the clinically healthy brother carried only the wild-type allele. The missense alteration was exclusive to the proband. **Conclusions:** This case exemplifies the significance of contemplating *TYK2* deficiency in pediatric patients with refractory mycobacterial infections, particularly in BCG-endemic locales. Genetic validation provided a definitive diagnosis, differentiating the condition from CGD and informing patient management. To our knowledge, this constitutes one of the inaugural genetically confirmed instances of *TYK2* deficiency in Central Asia, enhancing regional epidemiological comprehension and emphasizing the role of molecular diagnostics in directing treatment and vaccination policies.

## 1. Introduction

Inborn errors of immunity (IEIs), alternatively referred to as primary immunodeficiency disorders (PIDs), constitute a substantial and heterogeneous assemblage of genetic maladies characterized by compromised immune system development or functionality [1,2,3]. As of the present, over 500 distinct IEIs have been documented, with phenotypic expressions ranging from severe combined immunodeficiency to more selective cellular or humoral deficiencies [4]. These disorders are frequently recognized in early childhood due to recurrent, persistent, or atypical infections, autoimmune manifestations, or vulnerability to specific opportunistic pathogens [5]. Nonetheless, diagnosis is often postponed, particularly in resource-constrained environments, as clinical manifestations may coincide with prevalent infectious diseases or hematological conditions [6]. Significantly, individuals with IEIs are susceptible to life-threatening complications upon exposure to live-attenuated vaccines, such as Bacillus Calmette–Guérin (BCG), which is extensively utilized in tuberculosis-endemic areas. In these circumstances, heightened clinical awareness of IEIs is paramount [7]. Disseminated infection following routine vaccination may constitute the first indication of an underlying genetic defect. Timely recognition and genetic confirmation enable personalized patient management, genetic counseling for families, and informed decisions regarding immunization schedules and infection control strategies [8].

Among the heterogeneous spectrum of Inborn Errors of Immunity (IEIs), a distinct subset is characterized by Mendelian Susceptibility to Mycobacterial Disease (MSMD) [9]. These disorders are attributable to mutations in genes implicated in the interleukin-12/interferon-γ (IL-12/IFN-γ) signaling pathway, which is indispensable for host defense against intracellular mycobacteria [10]. Individuals with MSMD exhibit heightened susceptibility to infections by weakly virulent mycobacteria, encompassing environmental strains and BCG vaccine strains, in addition to more pathogenic *Mycobacterium tuberculosis* [11]. Clinically, affected persons may manifest localized or disseminated BCG infections subsequent to neonatal vaccination, presenting with lymphadenitis, osteomyelitis, pulmonary lesions, and cutaneous involvement [12]. These clinical presentations frequently resemble chronic granulomatous disease or recurrent bacterial abscesses, resulting in diagnostic ambiguity. In regions endemic for tuberculosis, where BCG vaccination is universally administered at birth, disseminated disease following vaccination strongly indicates an underlying immunodeficiency [13]. Notwithstanding its clinical relevance, MSMD remains underrecognized, particularly in Central Asia, where epidemiological data are sparse and access to sophisticated genetic testing has only recently become accessible.

One of the genes implicated in MSMD is *TYK2*, which encodes tyrosine kinase 2, a member of the Janus kinase family engaged in signaling for multiple cytokine receptors, including type I interferons, IL-12, and IL-23 [14]. Deficiency of *TYK2*, classified as Immunodeficiency-35 (IMD35), is an autosomal recessive disorder typified by increased vulnerability to mycobacterial and viral infections, recurrent respiratory disease, and in select cases elevated serum IgE levels [15]. Laboratory investigations frequently reveal normal quantities of immune cells but impaired cellular signaling responses, distinguishing *TYK2* deficiency from combined immunodeficiencies characterized by lymphopenia. Several pathogenic variants in *TYK2* have been documented, ranging from frameshift and nonsense mutations to missense substitutions impacting kinase functionality [16]. In certain patients, supplementary heterozygous variants in other immune-related genes, such as *MEFV*, which is associated with familial Mediterranean fever, may function as genetic modifiers contributing to variable phenotypic severity. The incorporation of whole-exome sequencing (WES) and segregation analysis has consequently become essential in identifying causative mutations and elucidating genotype–phenotype correlations in intricate cases [17].

Herein, we present the case of a Kazakh juvenile exhibiting recurrent disseminated BCG and tuberculosis infection, initially hypothesized to possess chronic granulomatous disease, but ultimately diagnosed with *TYK2* deficiency through WES and corroborative Sanger sequencing. The proband harbored two rare variants in *TYK2*, encompassing a pathogenic frameshift mutation and a missense substitution of uncertain significance, in conjunction with a heterozygous *MEFV* duplication variant. The clinical manifestation encompassed persistent lymphadenitis, osteoarticular and cutaneous tuberculosis, recurrent abscess-like lesions, and elevated immunoglobulin E (IgE) levels. The aim of this report is to elucidate the clinical trajectory, immunological characteristics, and genetic findings in this patient, thereby delineating the diagnostic pathway from clinical suspicion to molecular confirmation. This case underscores the significance of thorough immunological and genetic assessment in children with atypical or refractory mycobacterial infections, particularly in BCG-vaccinated cohorts. Furthermore, it contributes to the scant corpus of literature regarding *TYK2* deficiency from Central Asia, offering novel insights into regional genetic epidemiology. By disseminating this case, we aspire to broaden the clinical spectrum of *TYK2*-related immunodeficiency, accentuate the diagnostic complexities in distinguishing it from other granulomatous disorders, and highlight the pivotal role of molecular diagnostics in steering patient management and vaccination strategies in endemic regions.

## 2. Case Presentation

The proband was a female child born in late 2016 in the Aktobe region of Kazakh-stan, the second child of healthy, non-consanguineous parents. Pregnancy, delivery, and early developmental milestones were unremarkable. She received all routine neonatal vaccinations, including BCG at birth, without early complications. No family history of recurrent infections, autoimmune disorders, or known immunodeficiency or active or treated pulmonary tuberculosis was reported.

Until approximately 2 years of age, her medical history was notable only for occasional upper respiratory infections. Shortly after turning 2 years old, she developed an enlarging, tender mass in the left axillary region ipsilateral to the neonatal BCG vaccination site, accompanied by persistent fever. Surgical drainage was performed, but wound healing was prolonged, with ongoing serous discharge. Over the following months, she experienced recurrent abscess-like lesions of the axilla and anterior chest wall, together with progressive cervical and supraclavicular lymphadenopathy. Histology repeatedly showed nonspecific granulomatous inflammation without evidence of malignancy.

During her third year of life, she developed additional lesions in the anterior thoracic wall and neck, along with prolonged fever and general malaise despite antimicrobial and antituberculosis therapy. Imaging revealed multiple cystic and infiltrative lymph node and soft tissue lesions, raising concern for chronic granulomatous or disseminated mycobacterial disease.

In her fourth year of life, she was evaluated by a hematologist, and primary immunodeficiency was suspected. A trial of intravenous immunoglobulin (IVIG) was administered, but new lymphadenopathy and soft tissue lesions continued to appear. She was referred to national specialized centers, where she was diagnosed with generalized BCG/tuberculosis infection and restarted on first-line antituberculosis therapy. Partial clinical improvement was observed, though disease activity persisted. During the same year, she also developed acute appendicitis requiring surgical intervention.

By age 4–5 years, the child remained underweight but clinically stable. Examination revealed multiple scars over the axillae, cervical region, and anterior chest wall corresponding to prior surgical drainages and lymph node resections. No hepatosplenomegaly or acute respiratory distress was noted. The combination of disseminated mycobacterial infection, recurrent abscess-like lesions, and failure to respond to prolonged antituberculosis therapy raised strong suspicion for an inborn error of immunity. This prompted referral for comprehensive immunologic evaluation and WES.

## 3. Materials and Methods

### 3.1. Patient and Setting

This study concerns a single female pediatric patient born in November 2016 and evaluated between 2020 and 2021 at regional and national centers in Kazakhstan, including the National Scientific Center of Phthisiopulmonology and the “University Medical Center” Corporate Fund (Astana, Kazakhstan). The referral indication was recurrent, refractory lymphadenitis with multisite involvement and a clinical suspicion of primary immunodeficiency. Clinical data were abstracted from standardized hospital charts and consultation notes, including perinatal history, immunization record, prior procedures, growth and development, and longitudinal infectious episodes. Guardians provided written informed consent for clinical genetic testing and publication of anonymized data.

### 3.2. Patient and Setting Clinical Assessment and Imaging

A structured clinical examination was performed by pediatric infectious disease and immunology specialists. The assessment included general status, growth parameters, dermatologic survey for scars and draining sinuses, peripheral lymph node stations, respiratory system, and abdominal palpation. Radiology was obtained according to clinical need. Chest radiography was performed in standard projection. Chest computed tomography was performed with thin-slice acquisition and mediastinal and lung reconstructions to characterize lymphadenopathy and parenchymal findings. Soft tissue ultrasound was used to delineate subcutaneous collections and nodal architecture. Imaging was reviewed by board-certified radiologists.

### 3.3. Microbiology and Histopathology

Surgical material from affected lymph nodes and soft tissues was processed locally according to institutional protocols. Routine stains and histopathology were performed to differentiate nonspecific granulomatous inflammation from suppurative processes or malignancy. Mycobacterial cultures from lymph node tissue were positive for *M. tuberculosis* complex. Strain-level differentiation between *M. tuberculosis* and *M. bovis* BCG (e.g., PCR-based typing or pyrazinamidase testing) was not available at the treating institutions. Consequently, the diagnosis of BCG-related disease was established by national pediatric tuberculosis specialists based on the neonatal BCG vaccination history, chronic progressive lymphadenitis, and radiologic features typical of vaccine-derived infection. Bacteriologic evaluations and tuberculosis program diagnostics were conducted in accordance with national standards and pediatric TB guidelines.

### 3.4. Hematology, Inflammation, and Serum Immunochemistry

Complete blood counts and erythrocyte sedimentation rate were measured by automated analyzers with internal quality control, including a Sysmex XN-series hematology analyzer (Sysmex Corporation, Kobe, Japan). C-reactive protein and routine biochemistry were determined on clinical chemistry platforms, such as the Cobas c501 system (Roche Diagnostics, Mannheim, Germany). Total immunoglobulins (IgG, IgA, IgM) and complement components C3 and C4 were quantified by immunoturbidimetry or nephelometry according to manufacturer instructions, using reagent kits for BN II nephelometry (Siemens Healthineers, Erlangen, Germany). Total IgE and allergen panels were measured by immunoassay on an automated chemiluminescent analyzer (e.g., Immulite 2000, Siemens Healthineers, Erlangen, Germany). Viral serologies were performed by chemiluminescent or enzyme immunoassay per laboratory standard operating procedures, using commercial kits and platforms such as Architect i2000SR (Abbott Laboratories, Chicago, IL, USA).

### 3.5. Lymphocyte Immunophenotyping and Functional Assays

Peripheral blood lymphocyte subsets were quantified by flow cytometry using fluorochrome-conjugated antibodies against CD3, CD4, CD8, CD19, CD16, and CD56 with appropriate isotype controls, all purchased from BD Biosciences (San Jose, CA, USA). Flow cytometric acquisition and analysis were performed on a BD FACSCanto II flow cytometer (BD Biosciences, San Jose, CA, USA). Activated T cells were assessed with HLA-DR gating on CD3 positive lymphocytes. Absolute counts were derived from concurrent hematology indices.

Neutrophil oxidative burst was evaluated by a dihydrorhodamine-based assay (Phagoburst dihydrorhodamine 123 kit, BD Biosciences, San Jose, CA, USA) after phorbol ester stimulation with phorbol 12-myristate 13-acetate (PMA; Sigma-Aldrich, St. Louis, MO, USA) and reported as the percentage of reactive cells in granulocyte and monocyte gates. Phagocytosis assays for granulocytes and monocytes were performed with opsonized particles using manufacturer protocols, employing a commercial phagocytosis assay (e.g., Phagotest kit, BD Biosciences, San Jose, CA, USA). All assays followed internal controls and reference intervals validated by the clinical laboratory.

### 3.6. Whole-Exome Sequencing and Variant Calling

Genomic DNA was extracted from peripheral whole blood using a validated salt-out protocol, adapted from the QIAamp DNA Blood Mini Kit (QIAGEN, Hilden, Germany). Whole-exome sequencing libraries were prepared with a clinical exome capture kit, the SureSelectXT Clinical Research Exome (Agilent Technologies, Santa Clara, CA, USA), following manufacturer recommendations for fragmentation, end repair, adapter ligation, and enrichment. Paired-end sequencing was performed on an Illumina platform, specifically the NovaSeq 6000 system (Illumina Inc., San Diego, CA, USA). Base calling and FASTQ generation used the instrument software pipeline, bcl2fastq v2.20 (Illumina Inc., San Diego, CA, USA).

Reads were aligned to GRCh38 using BWA-MEM (v0.7.17; Broad Institute, Cambridge, MA, USA). Raw FASTQ files underwent quality control with FastQC (v0.12.1; Babraham Bioinformatics, Cambridge, UK). Post-processing included duplicate marking, base quality recalibration, and indel realignment following best practices using Picard tools (Broad Institute, Cambridge, MA, USA) and the Genome Analysis Toolkit (GATK v4.4.0.0; Broad Institute, Cambridge, MA, USA). Small variant discovery used a haplotype-based caller in gVCF mode (GATK HaplotypeCaller) with joint genotyping for family evaluation when parental samples were available.

### 3.7. Annotation, Filtering, and Clinical Interpretation

Variants were annotated with current releases of population and clinical databases and functional effect predictors, integrated through SnpEff (v5.2; Pablo Cingolani, Arlington, MA, USA) and Ensembl Variant Effect Predictor (Ensembl, Hinxton, Cambridge, UK). Population filtering removed common variation using global and ancestry-specific allele frequencies from gnomAD, 1000 Genomes, and other public repositories. Candidate variants were prioritized if exonic or canonical splice, predicted protein-altering, and located in genes consistent with mycobacterial susceptibility syndromes and primary immunodeficiencies. Classification followed ACMG and AMP guidelines, integrating criteria such as very strong loss-of-function evidence in disease genes, rarity, segregation, phenotype specificity, and supportive literature. Copy-number and structural variants were not systematically assessed. Final variant interpretation was performed in a multidisciplinary conference with correlation to clinical phenotype, immunophenotyping, imaging, and histology.

### 3.8. Sanger Confirmation and Segregation Analysis

Putative diagnostic variants were validated by bidirectional Sanger sequencing. Primers were designed to flank each locus by about 100 to 200 base pairs and synthesized at a national facility, the National Center for Biotechnology (Astana, Kazakhstan), using an ASM-800 DNA synthesizer (Biosset, Novosibirsk, Russia). PCR was performed under standard magnesium and cycling conditions with amplicon cleanup before capillary electrophoresis, using ExoSAP-IT (Applied Biosystems, Waltham, MA, USA) for PCR product purification. Capillary electrophoresis was conducted on an ABI 3500 Genetic Analyzer (Applied Biosystems, Waltham, MA, USA). Parental and sibling DNA was tested when available to assess inheritance (Table 1).

### 3.9. Ethical Compliance

The protocol, consent documents, and data handling procedures were approved by the Local Ethics Committee of the National Center for Biotechnology (Astana, Kazakhstan). The study conforms to the Declaration of Helsinki and national regulations. Written informed consent for genetic testing and publication of de-identified clinical information and images was obtained from the patient’s legal guardian.

## 4. Results

At the initial evaluation around age 3.5 years, the patient exhibited anemia, reactive thrombocytosis, and markedly elevated C-reactive protein (CRP), consistent with active systemic inflammation (Table 2). By the following year (around age 4.5 years), hemoglobin and platelet counts had improved, and CRP had declined substantially, although the erythrocyte sedimentation rate (ESR) remained persistently elevated (Table 2).

Serum studies in 2020 showed elevated IgG and a pronounced increase in total IgE, with IgA and IgM values slightly above or within reference intervals and normal C3 and C4 (Table 3). At follow-up in 2021, IgG had normalized, IgE had declined significantly, and complement levels remained stable and within normal limits (Table 3).

**Figure 1:**
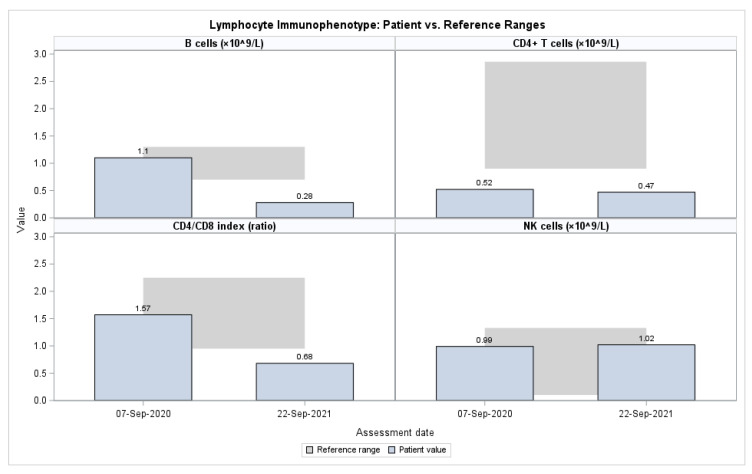
Lymphocyte immunophenotype in the patient compared with reference ranges.

Flow cytometry performed in 2020 and 2021 demonstrated persistently reduced CD4+ T cells and a low CD4/CD8 ratio, accompanied by a progressive decline in CD19+ B cells. In contrast, NK-cell proportions were consistently elevated at both time points, and activated T cells (CD3+HLA-DR+) increased from 6.1% to 14.9% (Table 4, Figure 1). Functional testing in 2020 revealed preserved neutrophil and monocyte oxidative burst responses and intact phagocytic capacity, all within reference ranges, effectively excluding chronic granulomatous disease as a cause of the patient’s clinical presentation (Table 5).

Serologic testing across 2020–2021 was negative for CMV, EBV, HSV-1/2, and hepatitis B. HIV testing was also negative. Eosinophil cationic protein (ECP) and total IgE were markedly elevated in 2020 and declined by 2021. Both adult and pediatric Phadiatop allergen panels were negative. Biochemical indices, including glucose, urea, and hepatic enzymes, remained within normal limits throughout the evaluation period.

Cross-sectional imaging during 2020–2021 revealed persistent mediastinal and peripheral lymphadenopathy and anterior thoracic wall lesions, consistent with disseminated mycobacterial infection. Histopathology of excised lymph nodes demonstrated nonspecific granulomatous lymphadenitis without evidence of malignancy. Mycobacterial cultures from lymph node tissue grew *M. tuberculosis* complex. Strain-specific molecular typing was not available; therefore, the infection was classified as BCG-related tuberculosis by the national pediatric TB team based on the documented neonatal BCG vaccination, the clinical pattern of chronic progressive lymphadenitis, and characteristic radiologic findings. The child had received a single BCG dose in the neonatal period.

WES identified two rare *TYK2* variants: a pathogenic frameshift mutation, *c.209_212del* (*p.Cys70Serfs21*), and a missense variant, *c.2395G>A* (*p.Gly799Arg*), previously reported in a Chinese patient with *TYK2* deficiency and functionally characterized as deleterious, classified here as a variant of uncertain significance in our ACMG-based interpretation (Table 6). A heterozygous MEFV duplication (*c.761_764dup*; *p.Asn256Argfs70*), classified as likely pathogenic, was also detected (Table 6). In population databases, *p.Gly799Arg* is absent or extremely rare, supporting its pathogenic potential; however, we classified it as a variant of uncertain significance according to ACMG criteria because no additional functional testing was performed in this patient.

**Figure 2:**
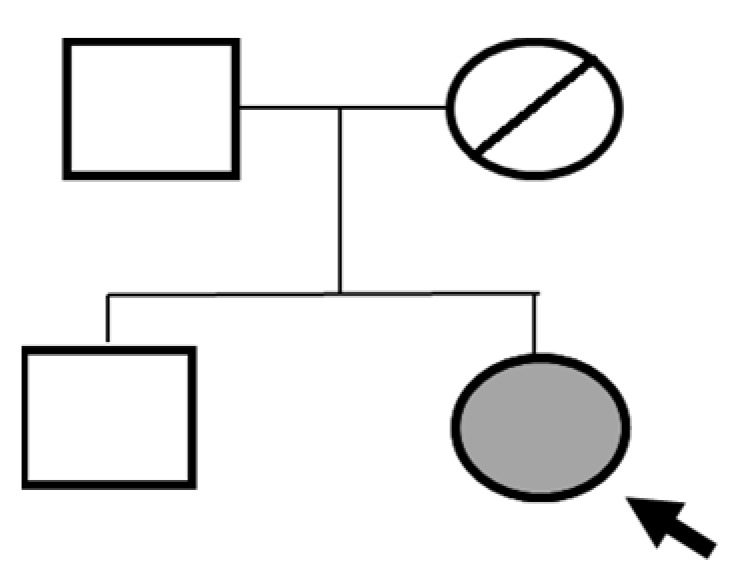
Pedigree of the proband with *TYK2* and *MEFV* variants, illustrating segregation in available family members. The mother is indicated as deceased by a diagonal slash through the symbol. The accompanying panel summarizes WES findings of the proband.

Sanger sequencing confirmed all three variants and clarified segregation within available family members (Table 7, Figure 2). The *TYK2* frameshift variant was present in the proband and mother, whereas the clinically and laboratory healthy brother carried only the wild-type allele. The missense *TYK2* variant was identified only in the proband. The *MEFV* duplication was found in all three individuals tested. Neither the proband’s mother nor brother had a history of recurrent fevers, serositis, or other symptoms suggestive of *MEFV*-associated autoinflammatory disease. Paternal DNA was unavailable; therefore, the allelic phase of the two *TYK2* variants (cis versus trans) cannot be experimentally confirmed, and this is acknowledged as a limitation of our study, although the overall genotype and phenotype are highly suggestive of autosomal recessive *TYK2* deficiency.

The proband’s brother, who is clinically and laboratorily healthy, did not carry the *TYK2 c.209_212del* frameshift variant. Paternal DNA was not available for analysis; phase of *TYK2* variants is inferred but not experimentally confirmed.

## 5. Discussion

The clinical course of this patient illustrates the classical yet variable manifestations of *TYK2* deficiency, a form of Mendelian Susceptibility to Mycobacterial Disease (MSMD) characterized by vulnerability to intracellular pathogens, particularly weakly virulent mycobacteria such as *Mycobacterium bovis* BCG [1,6,7]. Early-life dissemination of BCG following vaccination remains a hallmark presentation of IL-12/IFN-γ pathway defects [14,17]. From early childhood, our patient developed recurrent pulmonary and osteolytic infections, which were initially misattributed to complicated tuberculosis. Similar diagnostic delays have been documented in multiple cohorts due to overlapping clinical features with chronic granulomatous disease or severe tuberculosis [4,5,11,17,18,19,20]. The present case underscores the need for heightened clinical suspicion of IEIs when BCG-related infections persist or recur despite adequate antimicrobial therapy.

Molecular confirmation through whole-exome sequencing identified two rare *TYK2* variants, comprising a truncating loss-of-function allele and the *p.Gly799Arg* substitution, which has been previously reported in a Chinese patient and functionally validated as pathogenic in *TYK2* deficiency [18], strongly supporting *TYK2*-related MSMD in this child. However, because paternal DNA and *TYK2* cDNA cloning were unavailable, we cannot definitively determine whether the two variants are in trans, and we explicitly acknowledge this uncertainty regarding allelic phase as a limitation of our report. Functional studies in previous publications have shown that *TYK2* deficiency results in impaired IL-23–dependent induction of IFN-γ and defective downstream signaling of type I interferons [18,19]. No *TYK2* expression or cytokine-response assays were performed in our patient because of logistical constraints; therefore, we rely on previously published functional data for *p.Gly799Arg*. Although the precise immunophenotype of *TYK2* deficiency varies, most affected individuals exhibit normal lymphocyte counts but aberrant cytokine responses, distinguishing this condition from combined immunodeficiencies [4,5,17]. In our case, the immunological profile, featuring CD4+ lymphopenia and elevated IgE, corresponds to previously reported “hyper-IgE–like” phenotypes occasionally observed in *TYK2* deficiency [1,19].

The coexistence of an additional *MEFV* variant in our proband may act as a genetic modifier contributing to the inflammatory phenotype, as similar oligogenic patterns have been observed in MSMD and other IEIs [10,11,17]. The expanding use of next-generation sequencing (NGS) has uncovered such digenic and modifier interactions, revealing a more complex genetic architecture underlying immune dysregulation [11,20]. *MEFV*-associated autoinflammatory disease (familial Mediterranean fever) typically follows an autosomal recessive inheritance pattern, and single heterozygous carriers—such as the proband and her relatives—may remain clinically asymptomatic. In this context, we interpret the *MEFV* frameshift variant as a potential modifier with possible future relevance for reproductive counseling, rather than as a primary driver of the current phenotype, and we are cautious not to overstate its contribution. The inflammatory markers observed in our patient were largely consistent with a high burden of disseminated mycobacterial infection, and we did not observe *MEFV*-compatible autoinflammatory attacks in any family member. Segregation analysis in our family confirmed maternal carriage of the frameshift *TYK2* allele and absence of *TYK2* variants in the clinically healthy brother; paternal DNA was unavailable, so trans configuration cannot be formally proven, and this is explicitly acknowledged as a limitation of our study [1,11,19]. Importantly, these findings further broaden the global *TYK2* mutational spectrum, adding to reports from East Asia, Europe, and the Middle East [6,7,8,17,18,19]. To our knowledge, this case represents one of the first genetically confirmed *TYK2* deficiencies in Central Asia, contributing unique epidemiological data to an underrepresented region [15,16,21].

From a clinical perspective, this report reinforces the crucial role of early immunogenetic evaluation in children presenting with refractory or disseminated mycobacterial infections, particularly in BCG-vaccinated populations [6,11,13]. Delayed diagnosis, as observed here, perpetuates unnecessary procedures and prolonged anti-tuberculosis therapy without addressing the underlying immune defect [7,17,20]. Similar observations have been noted in the J Project registry, where diagnostic delays are common in low- and middle-income countries, including Uzbekistan and Kazakhstan, due to limited genetic testing capacity [15,16,21]. Earlier recognition of *TYK2* deficiency could have facilitated timely initiation of appropriate antimicrobial and immunomodulatory regimens, potentially reducing morbidity and preventing irreversible tissue damage. We acknowledge that strain-level confirmation of BCG versus *M. tuberculosis* by PCR or drug susceptibility testing was not available, which limits the precision of etiologic attribution and is a common diagnostic constraint in resource-limited settings.

On a broader scale, our findings highlight the pressing need to strengthen IEI diagnostic infrastructure in Central Asia and similar resource-limited settings. Recent registry data from Kazakhstan identified over 200 confirmed IEI cases between 2009 and 2023, yet most lacked molecular diagnosis until recent years [15]. These data echo earlier reports describing significant diagnostic barriers across the region, including lack of awareness, cost constraints, and absence of specialized immunology centers [16,21]. Collaborative initiatives such as the J Project have significantly expanded access to diagnostic and therapeutic support for IEI patients in 30 participating countries, emphasizing the transformative role of international cooperation in improving outcomes [21].

In this context, *TYK2* deficiency serves as a paradigm for the integration of genomic screening into public health and vaccination policy. Routine BCG vaccination, widely practiced in tuberculosis-endemic countries, poses a substantial risk for infants with undiagnosed IEIs [14,17,20]. Incorporating rapid genetic screening for key MSMD-associated genes (e.g., *TYK2*, *IL12RB1*, *IFNGR1*) in high-risk populations could mitigate these risks by enabling selective immunization strategies and targeted prophylaxis [11,13,17]. The recently proposed international guidelines on MSMD management advocate for early molecular diagnosis, genetic counseling, and careful consideration of live-attenuated vaccine use in suspected IEI cases [13,17,21].

Collectively, our report expands the clinical and molecular landscape of *TYK2* deficiency and provides region-specific evidence underscoring the necessity of genetic testing in patients with atypical mycobacterial disease. By aligning with global findings and regional epidemiological data, this study contributes to the broader effort to delineate genotype–phenotype correlations, refine diagnostic algorithms, and optimize care for rare primary immunodeficiencies [1,11,13,15,17,19,20,21,22].

## 6. Conclusions

This case demonstrates the diagnostic challenges posed by atypical and refractory mycobacterial infections in pediatric patients, particularly in BCG-vaccinated regions. The identification of two rare *TYK2* variants, supported by segregation analysis, established the molecular basis of the disease and explained the patient’s susceptibility to disseminated BCG/tuberculosis infection. The coexistence of an additional *MEFV* variant highlights the complexity of genetic backgrounds that may modulate clinical expression. Importantly, the preserved neutrophil oxidative burst and intact phagocytosis differentiated this condition from chronic granulomatous disease, underscoring the value of comprehensive immunological workup combined with genomic diagnostics. To our knowledge, this report represents one of the first genetically confirmed cases of *TYK2* deficiency from Central Asia, contributing novel insights into its regional epidemiology. Early recognition of such cases is crucial for optimizing patient management, guiding therapeutic decisions, and shaping vaccination safety policies in endemic settings.

## Figures and Tables

**Table 1:** Designed primers used for the validation of the identified variants.

Gene	Mutation	Primer Direction	Primer Sequence (5′ to 3′)
*TYK2*	*c.209_212del*	Forward	CTGGGAAATGTAGTCCTCCT
		Reverse	GCTCATACCTGCTCAAAGAG
*TYK2*	*c.2395G>A*	Forward	TGAGTACCTGAAGGGGACCTG
		Reverse	AGGCTGGGACTACATTGAG
*MEFV*	*c.761_764dup*	Forward	GCAGAAGGAGTGCAGGC
		Reverse	GGAATCACGCACACAGGTA

**Table 2:** Dynamic changes in hematologic and inflammatory markers.

Age					Parameters					
	Hemoglobin (g/L)	Reference Range	Leukocytes (×10^9^/L)	Reference Range	Platelets (×10^9^/L)	Reference Range	ESR (mm/h)	Reference Range	CRP (mg/L)	Reference Range
around 3.5 years	81	110–140	10.3	4.5–13.5	757	150–450	40	<20	121.98	<5
around 4.5 years	110	110–140	14.25	4.5–13.5	348	150–450	37	<20	25.40	<5

**Table 3:** Humoral indices.

Data								Humoral Indices			
	IgG (g/L)	Reference Range	IgA (g/L)	Reference Range	IgM (g/L)	Reference Range	Total IgE (IU/mL)	Reference Range	C3 (g/L)	Reference Range	C4 (g/L)
around 3.5 years	18.56	4.9–13.2	2.19	0.3–1.8	1.31	0.4–1.2	986.5	<60	1.10	0.75–1.65	0.29
around 4.5 years	13.80	4.9–13.2	0.95	0.3–1.8	2.07	0.4–1.2	68.43	<60	1.14	0.75–1.65	0.21

**Table 4:** Lymphocyte subsets.

Parameters	7 September 2020	22 September 2021	Reference
CD3+ T cells (%/×10^9^/L)	40.8/1.10	50.0/1.42	62–80/1.61–4.23
CD4+ T cells (%/×10^9^/L)	19.2/0.52	16.7/0.47	35–51/0.90–2.86
CD8+ T cells (%/×10^9^/L)	12.2/0.34	24.5/0.69	22–38/0.63–1.91
CD4/CD8 index	1.57	0.68	0.95–2.25
CD19+ B cells (%/×10^9^/L)	22.4/1.10	9.8/0.28	21–28/0.70–1.30
NK (CD16/56+) (%/×10^9^/L)	36.8/0.99	36.0/1.02	4–23/0.10–1.33
CD3+HLA-DR+ (%)	6.1	14.9	3–13

**Table 5:** Functional assays.

Test	7 September 2020	Reference
Neutrophil oxidative burst	97.1% reactive cells	97–100%
Monocyte oxidative burst	70.9% reactive cells	70–100%
Neutrophil phagocytosis	96.8%	95–99%
Monocyte phagocytosis	86.4%	65–95%

**Table 6:** Results of WES of the proband.

Variant Detected	Gene	Zygosity	Protein Change	Classification *
*c.209_212del*, *chr19-10368399 GAAGC>G*	*TYK2*	Heterozygous	p.Cys70Serfs*21	ACMG: Pathogenic (PVS1 + PM2 + PM3 + PP4); ClinVar: Pathogenic
*c.2395G>A*, *chr19-10357835 C>T*	*TYK2*	Heterozygous	*p.Gly799Arg*	ACMG: VUS (PM2 + PP4 + PP5); ClinVar: Pathogenic
*c.761_764dup*, *chr16-3254303 T>TGCGG*	*MEFV*	Heterozygous	p.Asn256Argfs*70	ACMG: Likely Pathogenic (PVS1 + PM2); ClinVar: Likely Pathogenic/VUS

* Classification according to ACMG/AMP guidelines and ClinVar database. For *TYK2*, two rare heterozygous variants were identified in the proband; paternal DNA was unavailable, so cis/trans configuration could not be established.

**Table 7:** Results of Sanger sequencing.

Gene/Variant	Sample	Genotype	Sequencing Data
*TYK2:c.209_212del* chr19-10368399 GAAGC>G p.Cys70Serfs*21	Proband	delCTTGC/CTTGC	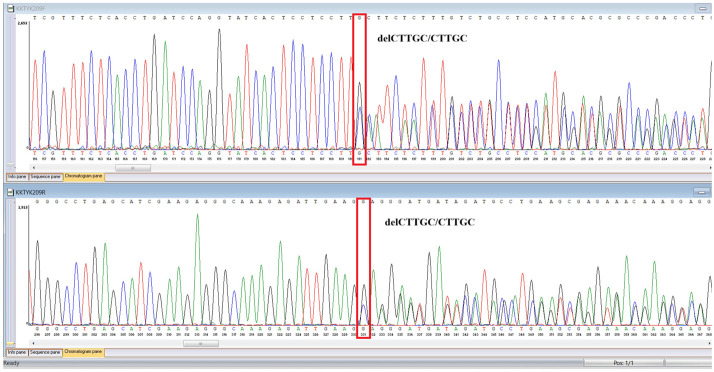
	Mother	delCTTGC/CTTGC	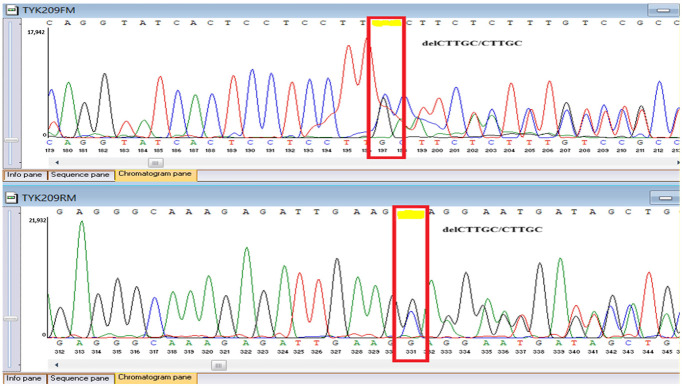
	Brother	CTTGC/CTTGC	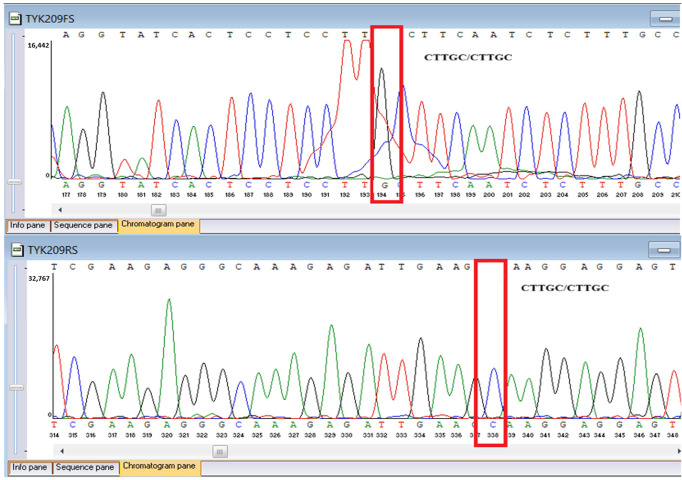
*TYK2:c.2395G>A* *chr19-10357835 C>T p.Gly799Arg*	Proband	G/A	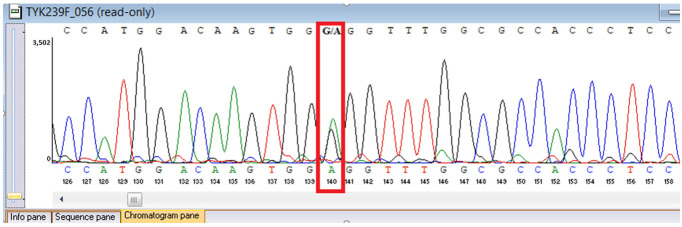
	Mother	G/G	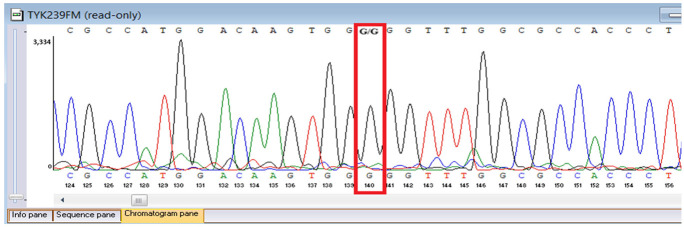
	Brother	G/G	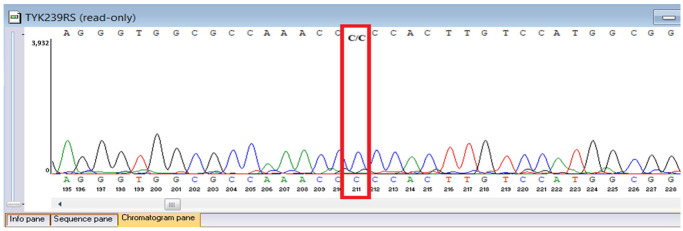
MEFV:c.761_764dup chr16-3254303 T>TGCGG p.Asn256Argfs*70	Proband	dupGCGG/GCGG	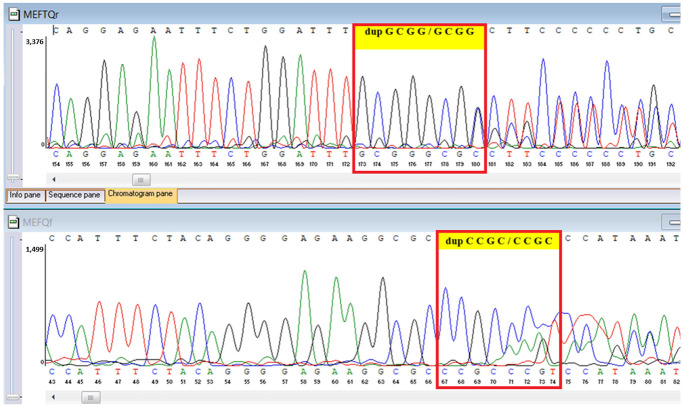
	Mother	GCGG/GCGG	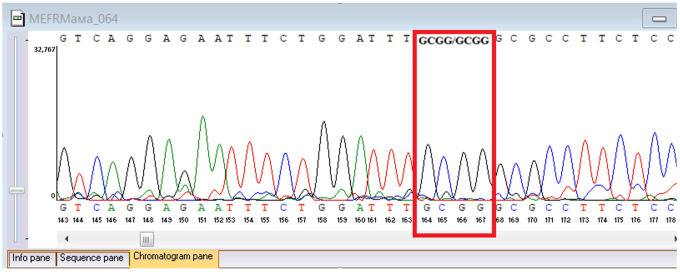
	Brother	GCGG/GCGG	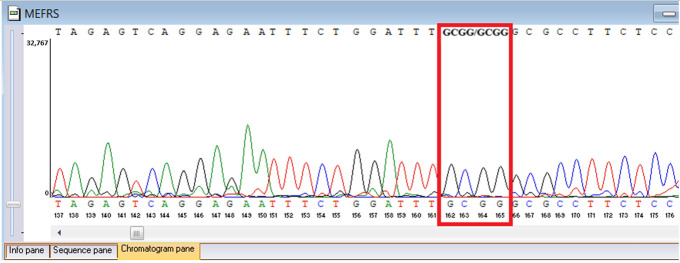

## Data Availability

The original contributions presented in this study are included in the article. Further inquiries can be directed to the corresponding author.

## Abbreviations

The following abbreviations are used in this manuscript:

ACMG	American College of Medical Genetics and Genomics
ADCL	Autosomal dominant cutis laxa
AMP	Association for Molecular Pathology
AR	Autosomal recessive
BCL	Base call file
CONSORT	Consolidated Standards of Reporting Trials
CT	Computed tomography
DNA	Deoxyribonucleic acid
dNTPs	Deoxynucleotide triphosphates
ELN	Elastin gene
Gdna	Genomic DNA
HGNC	HUGO Gene Nomenclature Committee
HGVS	Human Genome Variation Society
IEI	Inborn error of immunity
IUIS	International Union of Immunological Societies
MRI	Magnetic resonance imaging
NGS	Next-generation sequencing
PASP	Pulmonary artery systolic pressure
PCR	Polymerase chain reaction
PID	Primary immunodeficiency disorder
PICU	Pediatric intensive care unit
SNP	Single nucleotide polymorphism
SVAS	Supravalvular aortic stenosis
URDS	Urban–Rifkin–Davis syndrome
WES	Whole-exome sequencing
WGS	Whole-genome sequencing

## References

[1] Kreins A.Y., Ciancanelli M.J., Okada S., Kong X.F., Ramírez-Alejo N., Kilic S.S., El Baghdadi J., Nonoyama S., Mahdaviani S.A., Ailal F. (2015). Human TYK2 deficiency: Mycobacterial and viral infections without hyper-IgE syndrome. J. Exp. Med..

[2] Tangye S.G., Al-Herz W., Bousfiha A., Cunningham-Rundles C., Franco J.L., Holland S.M., Klein C., Morio T., Oksenhendler E., Picard C. (2022). Human Inborn Errors of Immunity: 2022 Update on the Classification from the International Union of Immunological Societies Expert Committee. J. Clin. Immunol..

[3] Bousfiha A., Moundir A., Tangye S.G., Picard C., Jeddane L., Al-Herz W., Cunningham-Rundles C., Franco J.L., Holland S.M., Klein C. (2022). The 2022 Update of IUIS Phenotypical Classification for Human Inborn Errors of Immunity. J. Clin. Immunol..

[4] Nemoto M., Hattori H., Maeda N., Akita N., Muramatsu H., Moritani S., Kawasaki T., Sugiura W., Yokomaku Y., Horibe K. (2018). Compound heterozygous TYK2 mutations underlie primary immunodeficiency with T-cell lymphopenia. Sci. Rep..

[5] Sarrafzadeh S.A., van de Veerdonk F.L., Paul W.E., Boisson-Dupuis S., von Bernuth H., Casanova J.-L. (2019). A new patient with inherited TYK2 deficiency. Front. Immunol..

[6] Wu P., Wang T., Zeng G., Wang J., Qi Y., Yu G., Ran Y., Li S., Liu Y., Hu Y. (2020). A TYK2 gene mutation c.2395G>A leads to TYK2 deficiency in a Chinese patient with BCG disease: Case report & review. Front. Pediatr..

[7] Guo W., Li Z., Cao X., Zhang Z., Li L., Yan Y., Zhang X. (2020). Mycobacterium intracellulare infection associated with TYK2 deficiency: Case report and literature review. Infect. Drug Resist..

[8] Xie L., Wang H., Zhou J., Li Y., Li H., Chen Y., Li Q. (2025). A new heterozygous TYK2 gene mutation: Case report and review of the literature. Int. J. Immunopathol. Pharmacol..

[9] NIH/NLM (2024). “Immunodeficiency 35 (TYK2 Deficiency)”—MedGen Summary. https://www.ncbi.nlm.nih.gov/medgen/409751.

[10] Yang Y., Xia L., Lu S. (2023). Adult-onset Mendelian Susceptibility to Mycobacterial Disease (MSMD): Expanding phenotypes and diagnosis challenges. Clin. Immunol. Open Access.

[11] Khavandegar A., Mahdaviani S.A., Zaki-Dizaji M., Khalili-Moghaddam F., Ansari S., Alijani S., Taherzadeh-Ghahfarrokhi N., Mansouri D., Casanova J.L., Bustamante J. (2024). Genetic, immunologic, and clinical features of 830 patients with Mendelian susceptibility to mycobacterial diseases: Insights from an international registry. J. Allergy Clin. Immunol..

[12] PanelApp (2025). Gene: TYK2 (Primary Immunodeficiency or Monogenic).

[13] Cornelissen H., Glanzmann B., van Coller A., Glashoff R., Goda R., Abdelmajeed O., Erwa N., Esser M. (2025). Clinical recommendations for diagnosis and management of Mendelian susceptibility to mycobacterial disease in resource-limited settings. J. Allergy Clin. Immunol. Glob..

[14] Filipe-Santos O., Bustamante J., Chapgier A., Vogt G., de Beaucoudrey L., Feinberg J., Jouanguy E., Boisson-Dupuis S., Fieschi C., Picard C. (2006). Inborn errors of IL-12/23- and IFN-γ-mediated immunity: Molecular, cellular and clinical features. Clin. Microbiol. Infect..

[15] Sikhayeva N., Kovzel E., Volodchenko S., Toleuzhanova A., Tortayeva G., Bukibayeva G., Zhussupbayeva Z., Morenko M. (2025). Registry-Based Frequency and Clinical Characteristics of Inborn Errors of Immunity in Kazakhstan: A Retrospective Observational Cohort Study (2009–2023). J. Clin. Med..

[16] Dauyey Z., Poddighe D. (2021). Diagnostic Barriers in Children with Immunodeficiencies in Central Asia: A Case-Based Discussion. Pediatr. Rep..

[17] Noma K., Mizoguchi Y., Tsumura M., Okada S. (2022). Mendelian susceptibility to mycobacterial diseases: State of the art. Clin. Microbiol. Infect..

[18] Ogishi M., Arias A.A., Yang R., Han J.E., Zhang P., Rinchai D., Halpern J., Mulwa J., Keating N., Chrabieh M. (2022). Impaired IL-23-dependent induction of IFN-γ underlies mycobacterial disease in patients with inherited TYK2 deficiency. J. Exp. Med..

[19] Roussel L., Pham-Huy A., Yu A.C., Venkateswaran S., Perez A., Bourdel G., Sun Y., Villavicencio S.T., Bernier S., Li Y. (2023). A novel homozygous mutation causing complete TYK2 deficiency, with severe respiratory viral infections, EBV-driven lymphoma, and Jamestown Canyon viral encephalitis. J. Clin. Immunol..

[20] Xia L., Liu X.-H., Yuan Y., Lowrie D.B., Fan X.-Y., Li T., Hu Z.-D., Lu S.-H. (2022). An updated review on MSMD research globally and a focus on under-recognized regions. Front. Immunol..

[21] Abolhassani H., Avcin T., Bahceciler N., Balashov D., Bata Z., Bataneant M., Belevtsev M., Bernatowska E., Bidló J., Blazsó P. (2022). Care of Patients with Inborn Errors of Immunity in Thirty J Project Countries between 2004 and 2021. Front. Immunol..

[22] Fazlollahi M.R., Nematollahi P., Pourakbari B., Mamishi S., Abolhassani H. (2024). Complications of the Bacillus Calmette–Guérin Vaccine as Early Clues to Inborn Errors of Immunity: A Report of 197 Patients. Front. Pediatr..

